# A deep-learning model to continuously predict severe acute kidney injury based on urine output changes in critically ill patients

**DOI:** 10.1007/s40620-021-01046-6

**Published:** 2021-04-26

**Authors:** Francesca Alfieri, Andrea Ancona, Giovanni Tripepi, Dario Crosetto, Vincenzo Randazzo, Annunziata Paviglianiti, Eros Pasero, Luigi Vecchi, Valentina Cauda, Riccardo Maria Fagugli

**Affiliations:** 1 Department of Applied Science and Technology, Politecnico Di Torino, C.so Duca degli Abruzzi 24, 10129 Turin, Italy; 2 Department of Electronics and Telecomunications, Politecnico Di Torino, C.so Duca degli Abruzzi 24, 10129 Turin, Italy; 3 Clinical Epidemiology and Pathophysiology of Renal Diseases and Hypertension, CNR-IFC, Nefrologia-Ospedali Riuniti, 89100 Reggio Calabria, Italy; 4 S.C. Nefrologia e Dialisi, Azienda Ospedaliera Di Terni, Viale Tristano Di Joannuccio, 05100 Terni, Italy; 5 S.C. Nefrologia e Dialisi, Azienda Ospedaliera Di Perugia, Piazzale Giorgio Menghini 1, 06129 Perugia, Italy

**Keywords:** Artificial intelligence, Acute kidney injury, EAlert, KDIGO

## Abstract

**Background:**

Acute Kidney Injury (AKI), a frequent complication of pateints in the Intensive Care Unit (ICU), is associated with a high mortality rate. Early prediction of AKI is essential in order to trigger the use of preventive care actions.

**Methods:**

The aim of this study was to ascertain the accuracy of two mathematical analysis models in obtaining a predictive score for AKI development. A deep learning model based on a urine output trends was compared with a logistic regression analysis for AKI prediction in stages 2 and 3 (defined as the simultaneous increase of serum creatinine and decrease of urine output, according to  the Acute Kidney Injury Network (AKIN) guidelines). Two retrospective datasets including 35,573 ICU patients were analyzed. Urine output data were used to train and test the logistic regression and the deep learning model.

**Results:**

The deep learning model defined an area under the curve (AUC) of 0.89 (± 0.01), sensitivity = 0.8 and specificity = 0.84, which was higher than the logistic regression analysis. The deep learning model was able to predict 88% of AKI cases more than 12 h before their onset: for every 6 patients identified as being at risk of AKI by the deep learning model, 5 experienced the event. On the contrary, for every 12 patients not considered to be at risk by the model, 2 developed AKI.

**Conclusion:**

In conclusion, by using urine output trends, deep learning analysis was able to predict AKI episodes more than 12 h in advance, and with a higher accuracy than the classical urine output thresholds. We suggest that this algorithm could be integrated in the ICU setting to better manage, and potentially prevent, AKI episodes.

**Graphic abstract:**

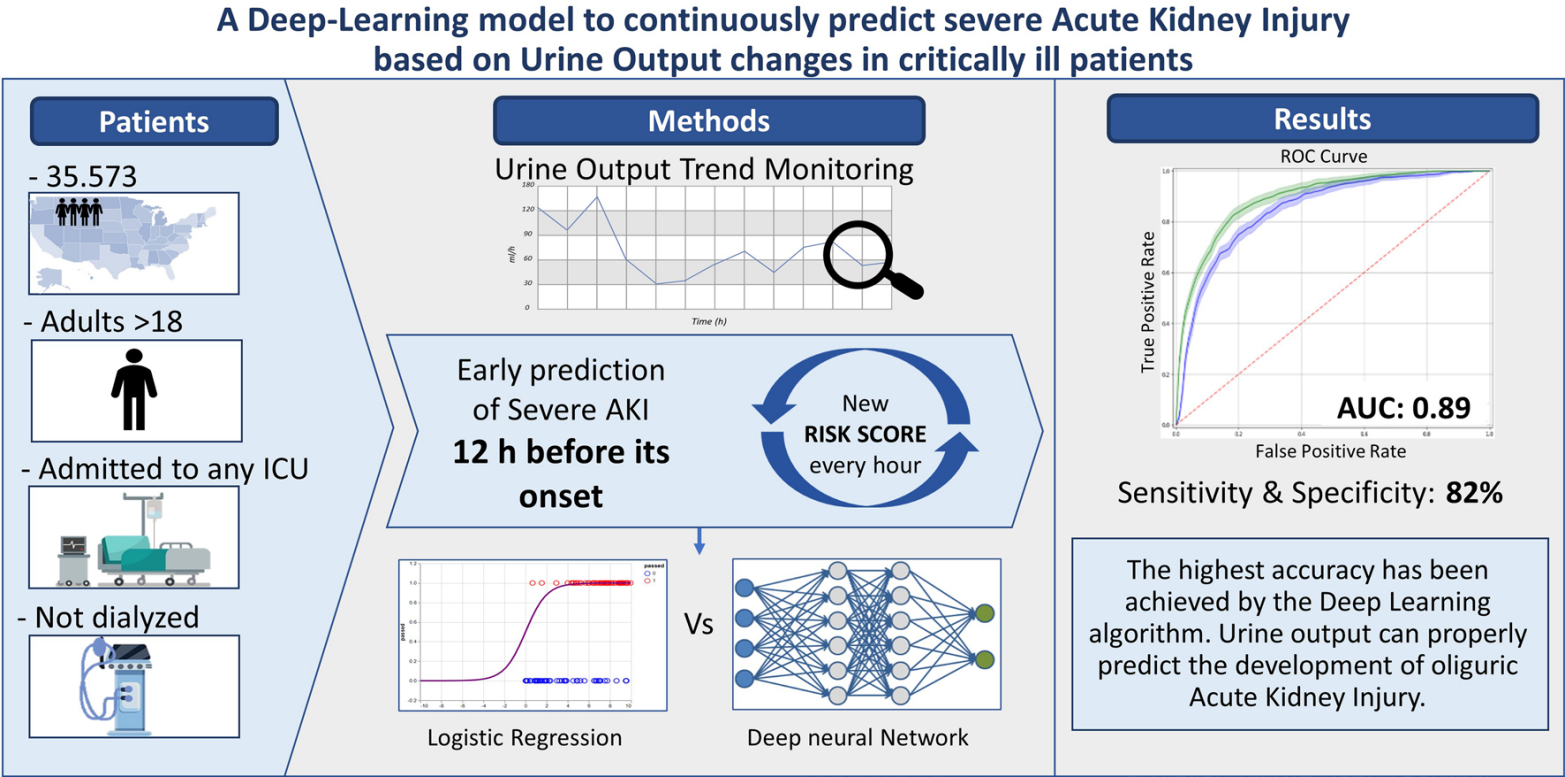

## Introduction

Acute kidney injury (AKI) is a global public health concern due to increasing patient complexities and aging populations. AKI patients do not only have prolonged periods of hospitalization and high mortality rates [1], but they also suffer from post-discharge, long term progression of kidney dysfunction [2] and mortality [3].

It is estimated that AKI occurs in about 13.3 million people/year, contributing to about 1.7 million deaths every year.

The International Society of Nephrology has promoted the 0by25 initiative for AKI, i.e. “zero preventable deaths by 2025” [4], in order to increase the awareness of AKI, to reduce the variations in its care, and to decrease preventable AKI deaths. The *“Recognition”* is a target of the care process, with both *“Diagnosis”* and *“Staging”* based on urine output (UO), serum creatinine (sCr), and new biomarkers.

Both the Acute Kidney Injury Network (AKIN) and the Kidney Disease: Improving Global Outcome (KDIGO) guidelines define oliguria as a reduction of urine output to < 0.5 ml/kg/hour [5]. Although about 60% of critically ill patients may have temporarily reduced urine output that is not necessarily linked to AKI, a reduction to < 0.5 ml/kg/h for a period of 6–12 consecutive hours is accompanied by a high need for dialysis and possible mortality [6]. The reduction of urine output to values of 1.8 ml/kg for 6 h also seems to have higher specificity on diagnosis than serum creatinine [7].

In view of the limitations linked to the urine output cut-off definition, a highly effective approach might involve the use of artificial intelligence (AI) and machine learning (ML). These techniques could replace the classical definition of urine output approach, and also increase both the sensitivity and specificity of AKI prediction.

The present study focuses on the use of deep structural learning, a part of machine learning based on artificial neural networks (ANN), which is believed to improve the care of patients and therefore the individual health outcome [8].

We retrospectively analyzed two large databases of critically ill patients, the electronic Intensive Care Unit (eICU) [9] and the Medical Information Mart for Intensive Care (MIMIC-III) [10], and we investigated the accuracy of logistic regression and of a deep learning model to predict the risk of AKI development with mathematical models based on urine output.

## Methods

### Study population

eICU and MIMIC-III databases are both available on PhysioNet. We analysed ICU patients older than 18 years, 38,597 patients of Beth Israel Deaconess Medical Centre (Boston), period 2001–2012 and 139,367 patients of 208 ICU, period 2014–2015. We included all ICU centers with more than 50 admissions per year, and after applying the exclusion criteria (Table 1), 35,573 patients were included.

**Table 1:** Exclusion criteria for a multi-center retrospective study of patients admitted to ICUs

Exclusion Criteria
Length of stay in ICU < 24 h
sCr baseline < 0.5 mg/dl
Community-acquired AKI
Patients undergoing dialysis during the ICU stay
Incomplete record of urine output (missing values for more than 9 h)
Incomplete record of serum creatinine (missing values for more than 4 days)
Patients from ICU centers with low activity volume (< 50 ICU admissions)

Serum creatinine, urine-output/hour and other additional information such as demographic characteristics, illness severity, and chronic comorbidities were extracted.

The study endpoint was defined as an “*AKI stage 2/3 AKIN*” episode, not requiring hemodialysis, defined by an increase in serum creatinine and a decrease in urine output.

Since baseline serum creatinine values were absent in both datasets that were used in the study, the creatinine baseline was calculated as the lowest reported ICU value. If a patient was admitted to the ICU twice or more, the lowest serum creatinine level was taken as baseline.

Urine output was normalized to ideal body weight (IBW) [13].

Patients with missing data for serum creatinine (> 4 days) and urine output (> 9 h), and patients from low volume ICU centers (< 50 ICU admissions) were excluded. Patients requiring dialysis were excluded from the analysis due to possible uncertain data of treatment initiation and cessation.

**Fig. 1:**
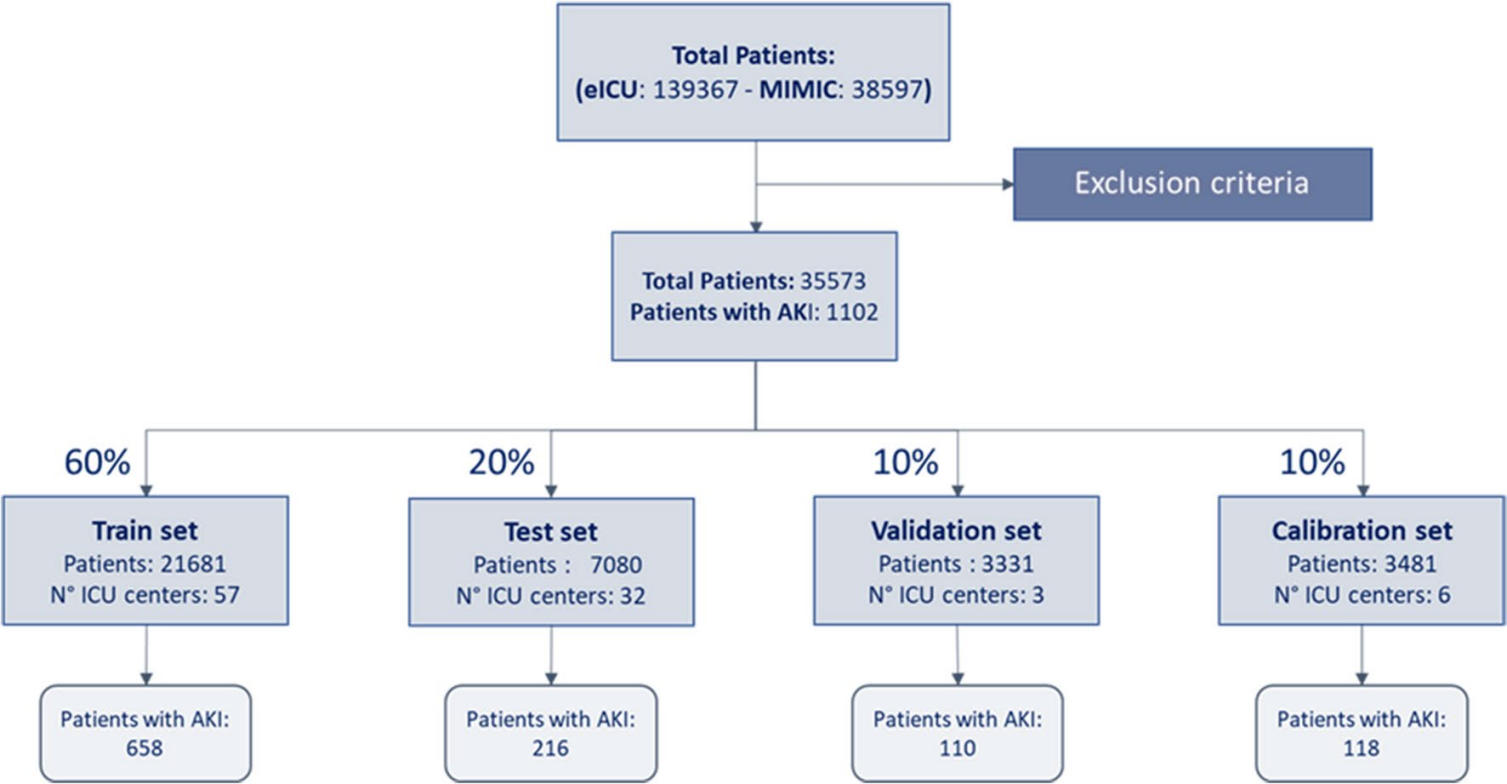
Patients remaining after exclusions and their distribution in splits

To conduct the mathematical analysis, data were randomly divided into four sets. The training set contains 60% of the data and was used to allow the model to observe and extract useful information (training phase). The validation set (10% of total data) was used to optimize the model’s parameters during the training phase, while the test set, containing 20% of total data, was used to assess the performance of the trained models. The last 10% (calibration set) was used for model calibration (Fig. 1).

### Definitions

#### Acute kidney injury

AKIN stage 2: serum creatinine increase from 200 to 300% and urine output < 0.5 ml/kg/h for a period > 12 h.

AKIN stage 3: serum creatinine increase higher than 300%, or equal to or greater than 4.0 mg/dl (≥ 354 μmol/l) with an acute increase of at least 0.5 mg/dl (44 μmol/l), and urine output < 0.3 ml/kg/h for a period > 24 h, or anuria for a period > 12 h.

#### Feature

**Fig. 2:**
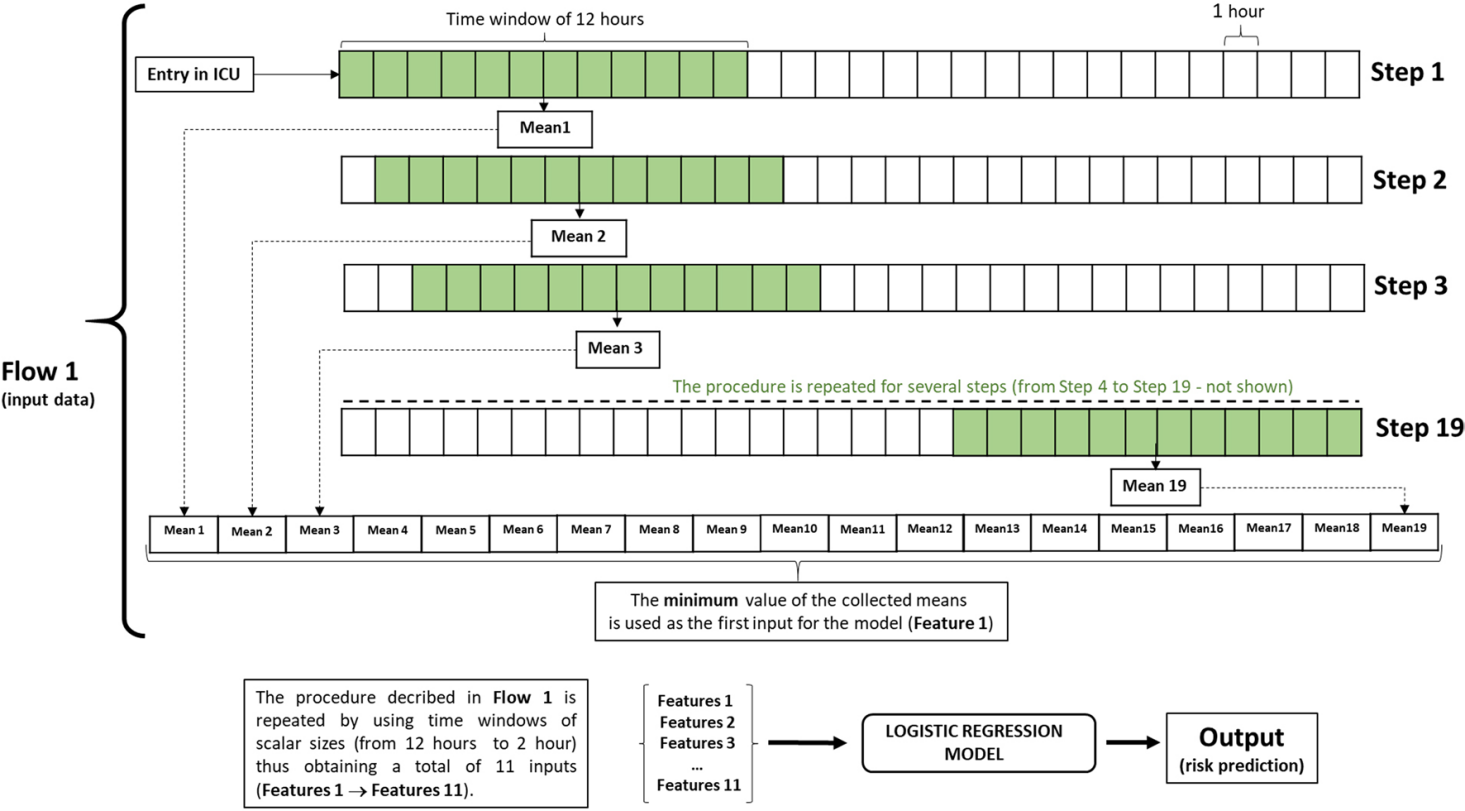
Example of extraction of the feature corresponding to a window size equal to 12. The process is repeated for all window sizes in range (2-12), thus obtaining 11 features

A feature is a peculiar attribute extracted from raw data. Features used in the current study were extracted from the hourly urine output trend of the patients and used for the logistic regression analysis. They are 11 values calculated as the minimum average values obtained by passing a series of sliding windows with a variable size in the range [2, 12] over the hourly urine output data from ICU admission to the hour of prediction (Fig. 2).

### Statistical analyses

Continuous variables were summarized as median and interquartile range and categorical variables as absolute numbers and percentages. The between-group comparison of cases (AKI stage 2/3) and controls was performed by Chi-Square Test or Mann–Whitney U test, as appropriate. The accuracy of the prediction model was assessed by ROC curve analysis as well as by calculating sensitivity, specificity, positive and negative likelihood ratios, and early detection percentage. Performances are evaluated at two different operation points: the first one is at 80% of sensitivity and the other is at knee-point which represents the best point reached by the ROC curve, close to the upper left bond.

### Data extraction and pre-processing

Baseline raw data of urine output and serum creatinine were extracted from the available databases (eICU and MIMIC-III). The collected data were manually recorded and entered by the nursing staff with variable sampling frequencies. 47% and 8% of total patients presented missing values in urine output and serum creatinine, respectively.

The data pre-processing phase required data transformation into time-series with a sampling rate of one hour. For serum creatinine, we dragged the value to the next available measurement if the gap between extracted values was < 4 days, while for urine output, data were imputed as follows: urine volume for a time interval without recording (if the gap was < 9 h) was included in a cumulative total which was sub-sequentially divided by the number of hours in the time interval and assigned to each hour.

### Mathematical models

Two separate analyses were conducted both using as a starting point the hourly urine output of patients normalized for their ideal body weight (ml/h/kg) calculated according to Devine’s formula (see Appendix) [13].

For patients with no episodes of AKI (2 or 3 AKIN), the time series were used entirely from entry into the ICU until discharge or death. The time series of patients with AKI onset (2 or 3 AKIN) episodes labeled as ‘case’, were truncated 6 h before the event to ensure the prediction at least 6 h before the event.

The data were split into training, validation, calibration and test sets so that information from a given patient was present only in one split.

### Logistic regression model

This model, as described elsewhere [7], uses some distinctive characteristics extracted from data named ‘*features’* and takes those *‘*features’ as input of the model. After a mathematical computation, the model outputs the probability of developing AKI stage 2/3.

In this study, the ‘features’ are extracted from the hourly urine output trend of the patient within the ICU stay. A series of sliding windows with a variable size from 2 to 12 h were used to compute the moving average along with the hourly urine output patients’ time series. Later, the minimum value of the averages for each sliding window was used as a feature. This process was repeated for all hours of the ICU stay, starting from the 12th hour since admission until the occurrence of the AKI episode or ICU discharge (Fig. 2). To analyze truly independent samples, only one set of features per patient was randomly selected.

Two analyses were conducted with the extracted features: one required the use of all available features, while the other used a single feature each time.

In both cases, the models were trained on 10-randomly selected training sets and performances were evaluated on 100 randomly selected independent test sets.

### Deep learning model

**Fig. 3:**
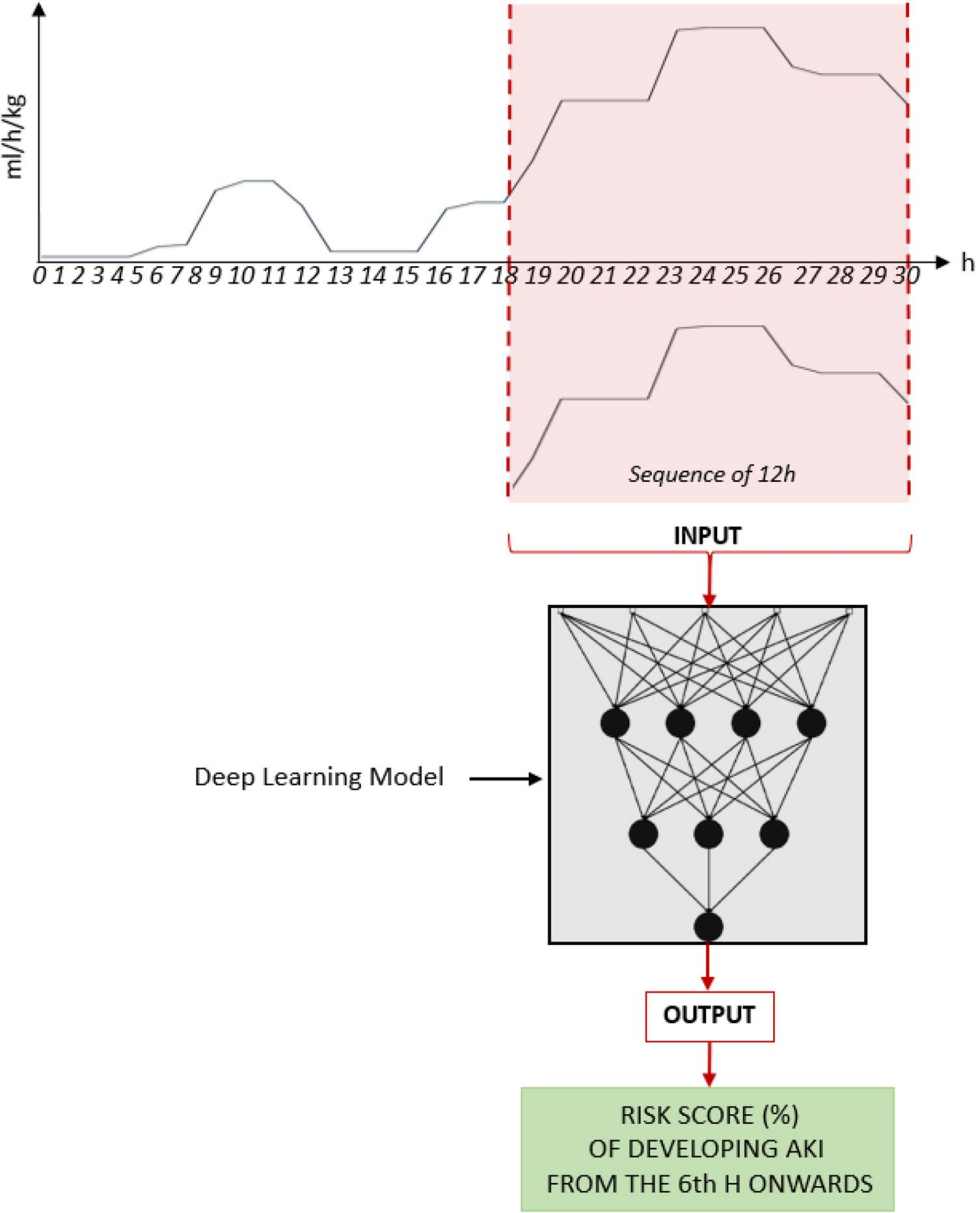
Example of a deep learning model during an ICU stay. The urine output trend shown above belongs to a patient admitted to the ICU for 30 h. At the 30th hour, a new risk score of developing severe AKI from 6 h onwards is generated by the deep learning model using the last 12 h of urine output data as input

Sequences of 12 h of hourly urine output recordings were used as input of our deep learning model. The output of this model corresponds to the probability of developing AKI stage 2/3 starting from the sixth hour from prediction onwards. The risk probability is updated every hour during the ICU stay (Fig. 3).

An iterative approach was used to train a model across 10 randomly selected training sets. The validation set was used to improve the model by selecting the best model architectures and hyperparameters. To evaluate its performances, 100 randomly selected independent test sets were used, the results are shown averaged.

The deep learning model core is constituted of stacked and parallel layers of convolutional neural networks (CNNs) with highway connections between each layer. The main advantage of using a deep architecture is implicit in its ability to automatically extract features from raw data and understand which are useful for solving that specific problem. We chose a convolutional neural network from a broad range of alternative architectures thanks to its demonstrated ability to match with time series classification problems [14]. Raw time series were processed by the network with appropriate types and combination of layers able to extract the prominent and representative characteristics of data and enhance the learning.

## Results

We included 21,681 patients in the training cohort, 7080 patients in the test group, 3331 patients in the validation one and 3481 in calibration split. Among the groups, the percentage of patients with an episode of AKI stage 2 or 3 is approximately the same: 3%.

The median age of the 35,573 patients involved in our study was 67 (Interquartile range [IQR]: 55–77) years, 62.7% were males, 2.1% were diabetics (diabetes mellitus type 2), 8.6% had pre-existing chronic kidney disease (CKD) and 7.7% had cardiac disease. Around 3% of the patients developed AKI, and 30% of them died during hospitalization (Table 2).

**Table 2:** Summary statistics for the data

	Total	AKI 2/3	Others	*p*-Value
Patients	35,573	1102	34,471	–
Gender M	22,331 (62.7%)	711 (64.5%)	21,620 (62.7%)	0.248
Average hospital stay length	67.35 h (44.3–112.2)	169.23 h (96.6–290.1)	67.72 h (43.7–106.1)	< 0.001
Average age	67 (56–77)	68 (57–78)	67 (56–77)	< 0.001
CKD	3066 (8.6%)	107 (9.7%)	2959 (8.6%)	0.485
DM type II	754 (2.1%)	15(1.4%)	739(2.1%)	0.050
Heart Disease	2,743 (7.7%)	111 (10.0%)	2632 (7.6%)	0.026
Death	2,171 (6.1%)	336 (30.5%)	1835 (5.3%)	
Min diuresis value (ml/hr/kg)	0.29 (0.16–0.47)	0.10 (0.05–0.18)	0.29 (0.16–0.48)	< 0.001
Max diuresis value (ml/hr/kg)	4.41 (2.53–6.92)	2.15(1.03–4.44)	4.47(2.59–6.98)	< 0.001
Min serum creatinine value	0.8 (0.63–1.03)	0.9 (0.7–1.2)	0.8 (0.63–1.02)	< 0.001
Max serum creatinine value	1.10 (0.84–1.50)	2.04 (1.52–2.80)	1.06(0.83–1.43)	< 0.001

*CKD* Chronic Kidney Disease; *DM* Diabetes Mellitus

Patients in the four sets used for the analysis (Table 3) show no relevant differences in terms of average age, gender and average hospital stay length. It should be noted that there are some differences in the presence of co-morbidities such as chronic kidney disease, heart disease and diabetes. This might be due to the random division of patients into sets.

**Table 3:** Summary statistic for sets

	Training set	Test set	Validation set	Calibration set
Patients	21,681	7,080	3,331	3,481
Gender M	13,747 (63.4%)	4,476(63.2%)	2,019 (60.1%)	2,089 (60.0%)
Average hospital stay length	68 (45–115)	65.34 (45–107)	64.92 (42–102)	64.52 (41–111)
Average age	68 (57–78)	67 (56–78)	68 (55.5–77)	66 (54–76)
CKD	1,816 (8.4%)	726(10.2%)	240 (7.2%)	284 (8.1%)
DM type II	324 (1.5%)	303(4.3%)	56 (1.7%)	71 (2.0%)
Heart Disease	1,344 (6.2%)	841 (11.9%)	285 (8.5%)	273 (7.8%)
Min diuresis value (ml/hr/kg)	0.29 (0.16–0.46)	0.29 (0.15–0.49)	0.26(0.12–0.46)	0.31(0.16–0.49)
Max diuresis value (ml/hr/kg)	4.68 (2.73–7.17)	3.92( 2.33–6.29)	4.21 (2.23–6.93)	3.86 (2.26–6.51)
Min serum creatinine value	0.8 (0.62–1.02)	0.8 (0.64–1.05)	0.8 (0.62–1.00)	0.78 (0.63–1.05)
Max serum creatinine value	1.1 (0.84–1.5)	1.07(0.84–1.50)	1.09(0.84–1.5)	1.07(0.83–1.55)
AKI stage 2/3	658 (3.0%)	216 (3.0%)	110 (3.3%)	118 (3.4%)
In-Hospital Death	1,402 (6.5%)	410 (5.5%)	183 (5.5%)	199(5.7%)

*CKD* Chronic Kidney Disease, *DM* Diabetes Mellitus

### Logistic regression model

**Fig. 4:**
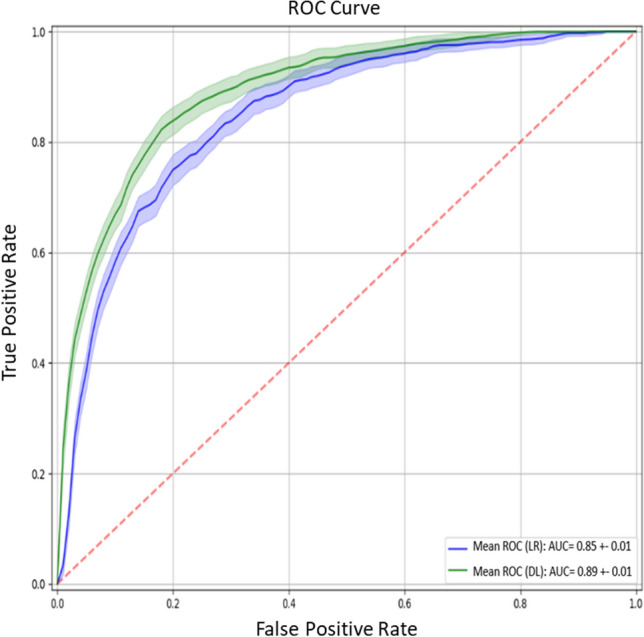
Receiver operating characteristic curve for the acute kidney injury prediction model.* AUC* rea under the receiver operating characteristic curve

The multi-feature analysis conducted with the logistic regression model and evaluated on the test set reached an area under the ROC curve of 0.85 (Fig. 4). Specificity was 78% while sensitivity was equal to 77% in the test group by considering performances at the knee-point (threshold = 56), while the same model at fixed 80% (threshold = 70) of sensitivity proved to be 75% specific. In terms of time of prediction, the logistic regression model predicted 79% of AKI cases by 12 h before they met the diagnostic criteria. The current model gives a positive likelihood ratio (at 80% of sensitivity) of ~ 3 and a negative likelihood ratio of ~ 0.3 (Table 4). To put these results in perspective, for every 4 AKI triggered alarms, 3 are effectively running into high risk of developing AKI and one is a false alarm. As for the negative likelihood ratio, for every 13 non-triggered alarms 3 are false negatives, while 10 have a real low to null risk of AKI.

**Table 4:** Numerical results for the multi-feature logistic regression model

Model	Working point	auROC (avg)	Sensitivity	Specificity	LR +	LR-
Logistic Regression	Sensitivity = 80%	0.85 ± 0.01	80.0%	75.0 ± 2.6%	3.20	0.31
	Knee-point		77.4%	78.0 ± 2.9%	3.52	0.29

*auROC* area under receiving operator curve *LR +* likelihood positive ratio, *LR-* likelihood negative ratio

From the single-feature analysis, we obtained the results shown in Table 5.

**Table 5:** Numerical results for the single-feature logistic regression model

Window (h)	Threshold (ml/h/kg)	Sensivity	Specificity	Precision	LR+	LR−	auROC
2	0.251	0.733	0.733	0.008	2.745	0.364	0.786
3	0.288	0.733	0.733	0.008	2.745	0.364	0.786
4	0.311	0.739	0.741	0.009	2.853	0.352	0.798
5	0.341	0.738	0.741	0.01	2.849	0.354	0.804
6	0.362	0.739	0.741	0.011	2.853	0.352	0.810
7	0.372	0.755	0.759	0.012	3.133	0.323	0.813
8	0.407	0.743	0.741	0.012	2.869	0.347	0.817
9	0.427	0.749	0.75	0.012	2.996	0.335	0.815
10	0.457	0.744	0.741	0.013	2.873	0.345	0.817
11	0.471	0.75	0.75	0.012	3	0.333	0.818
12	0.487	0.753	0.75	0.013	3.012	0.329	0.817

It can clearly be observed that by adopting the single-feature approach, the area under the ROC curve for any window-length leads to the worst performance rather than the ones obtained by using the multi-feature and the deep learning approaches. The best result achieved corresponds to the features extracted with a window-length of size 7, which generates an area under the ROC curve and a positive likelihood ratio equal to 0.81 and 3.1, respectively. It was 75% sensitive and roughly 76% specific. The latter model uses a threshold to discriminate between AKI and non-AKI cases, identifying patients whose average urine output in a time interval of 7 h remains below 0.372 ml/kg/h as being highly exposed to AKI.

### Deep learning model

After assessing the performances of the deep learning model on the test set, we obtained levels of sensitivity and specificity of 82% (threshold = 70) at the knee-point with an area under the ROC curve of 0.89 (Fig. 4). To better compare the performances of our two explored models, we chose a sensitivity score of 80% (threshold = 72) and extracted a sensitivity of 84%. The deep learning model was able to predict 88% of AKI cases 12 h before they met the diagnostic criteria. This model reaches a high positive likelihood ratio [5] and a small negative likelihood ratio (0.2) (Table 6). Practically speaking it means that for every 6 AKI alarms that were triggered, 5 resulted true and only one was a false alarm. Alternatively, only 2 cases out of 12 non-triggered alarms are false negatives, while the remaining 10 are actually at low risk of developing AKI. The predictive value did not differ significantly for AKI stage 2 and stage 3, as is reported in Table 7.

**Table 6:** Numerical Results for the Deep learning Model

Model	Working point	auROC (avg)	Sensitivity	Specificity	LR+	LR-
Deep Learning	Sensitivity = 80%	0.89 ± 0.01	80.0%	84.0 ± 3.0%	5.00	0.20
	Knee-point		82.0%	82.0 ± 3.0%	4.50	0.22

*auROC* area under receiving operator curve *LR +* likelihood positive ratio, *LR-* likelihood negative ratio

**Table 7:** Predictive value for AKIN stage 2 and 3

Model	Stage AKI	auROC	Sensitivity	Specificity	LR+	LR-
Deep Learning	2	0.89	80.0%	83.6%	4.87	0.24
	3	0.89	83.0%	83.6%	5.06	0.20

*auROC* area under receiving operator curve *LR +* likelihood positive ratio, *LR-* likelihood negative ratio

**Fig. 5:**
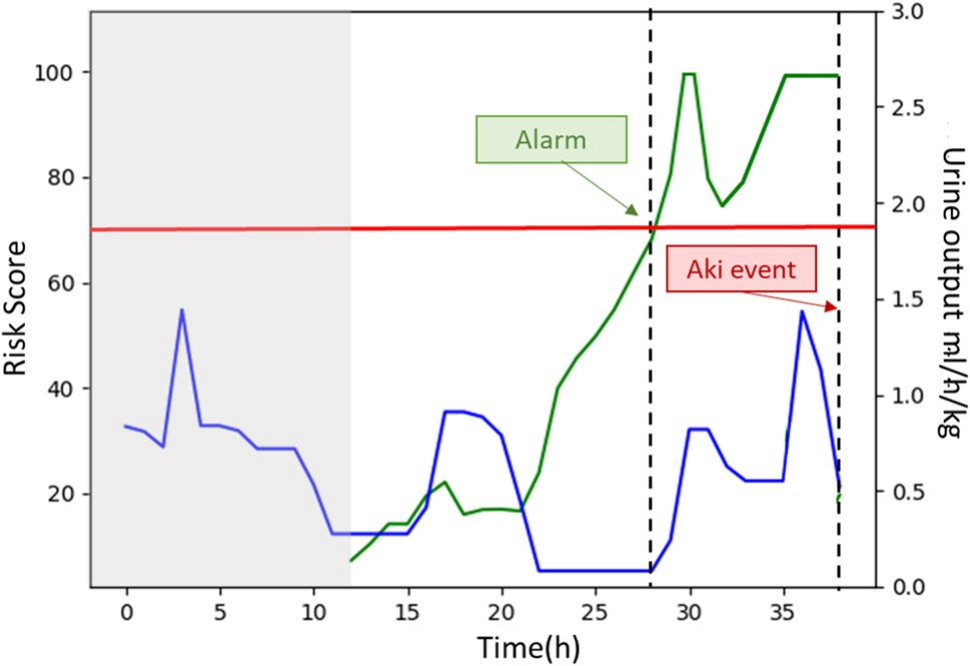
Example of the urine output trend of a patient with AKI stage 2 and 3 AKIN (solid blue line) and an example of prediction model output (solid green line). The red horizontal line corresponds to the threshold used to trigger the alarm

The AKI prediction model produces a continuous score from 0 to 100, representing the probability of risk of developing AKI. All scores above 70% indicate a high risk while scores below 70% indicate low risk. This score is not strictly related to the single hourly value of urine output but is the result of a more complex computation made by the deep learning model, which provides the urine output trend during the previous 12 h. An example is shown in Fig. 5 where the risk score is given after the first 12 h of the patient’s ICU stay.

## Discussion

Acute Kidney Injury is a relevant event that pervades health care systems and has poor outcome. Although the burden of AKI varies depending on its classification, on the use of administrative or clinical data [15], on the clinical setting and on differences between high and low resource countries, an increase in AKI episodes has been observed over the last two decades: a study conducted in England between 1998 and 2013 reported that hospital-acquired AKI not requiring dialysis increased from 317 to 3995 cases per million population (pmp) [16]. In the cases of AKI requiring dialysis (AKI-D), a retrospective study conducted in the USA demonstrated an increase in episodes from 222 to 533 pmp between 2000 and 2009 [17]. In the ICU, critically ill patient AKI incidences varied from 10 to 50% [18–21].

AKI is linked to a high mortality rate, ranging from 10 to 60%, depending on its severity and the concomitant failure of other organs. Surviving patients may develop decreased renal function, need for chronic dialysis treatments and an increased risk of cardiovascular disease and subsequent mortality [22].

According to the AKIN guidelines, a serum creatinine increase (AKI-sCr), *“or”* a urine output decrease (AKI-Uo), defines the presence and relevance of AKI [12]. When urine output is considered, whether or not it is associated with an increase in serum creatinine, a higher number of AKI cases are diagnosed, and the detection can be made earlier [19]. The duration of oliguria appears to be associated with the beginning of dialysis and an increased risk of death [23]. The definition of oliguria has recently received critical appraisal: the impact of both the volume of urine as well as the duration of oliguria on AKI prediction is controversial: KDIGO Clinical Practice Guidelines for Acute Kidney Injury [24] define a urine output < 0.5 ml/kg/h for > 6 h as AKI stage 1, < 0.5 ml/kg/h for > 12 h as AKI stage 2, and a < 0.3 ml/kg/h for 24 h or anuria for 12 h as AKI stage 3.

Prowle et al. reported that only 15% of ICU patients with an episode of oliguria developed AKI-sCr stage 2. If oliguria persisted for at least 12 h, the relative risk of developing AKI-sCr the following day was equal to 11.5 with a positive likelihood ratio of 13.5 [25]. Macedo et al. observed oliguria before serum creatinine increase, and this allowed an earlier diagnosis of AKI stages 2 and 3: 6 consecutive hours with UO < 0.5 ml/kg/h were linked to the highest rate of progression to AKIN-sCr stage 2 (79%). A urine volume of less than 0.72 ml/kg/h for 24 h was able to predict AKIN-sCr stage 3 with positive and negative values of 0.37 and 0.76 [6]. Ralib et al. observed that a 6-h urine output threshold of 0.3 ml/kg/hour best-predicted mortality and need for dialysis, with a positive and negative predictive value of 0.34 and 0.90, instead of 0.28 and 0.89 as reported for serum creatinine [7]. The prospective FINNAKI study [38] reported an increase in AKI-sCr risk for a 3–6 h period of urine output < 0.1 ml/kg/h and an increased risk of mortality at 90 days.

We investigated the predictive value of urine output on AKI stage 2/3 using two large databases of patients admitted to ICUs, the eICU and the MIMIC-III [9, 10].

In the case of *“big data”*, the information can be elaborated mathematically with analytic methodologies. With regard to medicine, the use of artificial intelligence has been significantly developed in two branches: physical and virtual. The virtual branch consists of machine learning methods, characterized by algorithms and statistical models that learn from data that are able to recognize and deduce patterns.

Our study applies a machine learning method to the determination of the risk of AKI development considering urine output, and consequently evaluating the algorithm to define an electronic alert. Here, two methods were used and compared: the deep learning model obtained the highest sensitivity and specificity of 82% with an AUC of 0.89. The accuracy of this model was confirmed with the high positive and small negative likelihood ratio. The performance of deep learning appears to be superior to the logistic regression model. Logistic regression reveals a likelihood positive ratio of 3.13 and a likelihood negative ratio of 0.32, with a urine output threshold of 0.37 ml/kg/h for 7 h.

The deep learning model differs from the previous method as it does not consider urine output in terms of volume and time, but it analyzes the dynamic changes: the result is the higher accuracy of the predictive score, with the highest positive likelihood ratio equal to 5 and the lowest negative likelihood ratio equal to 0.2.

The deep learning model was able to predict 88% of AKI cases at least 12 h before the event: for every 6 patients identified as being at risk of AKI by the deep learning model, 5 experienced the event. For every 12 patients not considered to be at risk by the model, 2 developed AKI.

AI can analyze the relationship between *“big data”* as *“input”* and *“events”* as *“output”*.

Concerning AKI prediction in the ICU, the study of Huang et al. analyzed 9,791 MIMIC III and eICU patients [27] and investigated the predictive value of 52 clinical sets of data collected over 6 h. The data included 8 classes, such as fluid balance, demographic, anamnestic, clinical interventions, and laboratory results. The AdaBoost predictive algorithm revealed the highest AUC = 0.88 for AKI-sCr and/or AKI-Uo occurrence during the first week of an ICU stay.

Flechet et al. [28] analyzed the performance of the prediction model for the development of AKI in the ICU by using data from the retrospective study of EPaNIC. Data used for the prediction models were based on information obtained at ICU admission and after the first 24 h, and also included the total amount of urine and the slope. The outcome was AKI at any stage or AKI at stages 2 or 3, which manifested during the first week of an ICU stay. The predictive performance was also compared with mathematical models with a biochemical marker such as serum neutrophil gelatinase-associated lipocalin (NGAL) levels. The performance of the mathematical model was high, with an AUC of 0.84, which was higher than NGAL with an AUC of 0.74.

Zimmermann et al. [29] obtained different results using data from MIMIC-III. The input data regarded variables recorded during the first day of an ICU admission including gender, age, heart rate, blood pressure, SpO2, lab values and the hourly rate of urine output. The outcome was AKI at any stage on days 2 and 3 of an ICU stay. Univariate analysis did not show a significant association between urine output and continuous creatinine outcome.

These studies differ from our investigation because of the endpoint. AKI development is similar but the time is different: the first week of the ICU stay in the study of Huang and Flechet, and days 2 ad 3 in the study of Zimmermann. In our study, urine output is entered as continuous data during the entire ICU stay, (as the output AKI stages 2 or 3).

Our study can be applied to a generation of electronic Acute Kidney Injury alert systems (eAKI) which enable earlier detection of AKI. Some warning systems [31], but not all [32], have proven to be of benefit to the patients in terms of diagnostic procedures, treatment, and nephrology consultation [33]. Park observed that eAKI permitted a reduction in the severe progression of renal failure and an improvement in AKI recovery, but failed to report the effects on mortality [34]. Prendecki reported a better outcome in terms of survival and need for renal replacement therapy [30].

Our study triggers the calculation of a risk score obtained with continuous input of data, urine output measured as ml/kg/h, with the ability to “alert” the medical staff 12 h before the onset of AKI.

The impact of electronic alerting of AKI essentially results in the possibility of earlier medical intervention from the diagnosis of changes in clinical conditions which could be responsible for decreased kidney function (for example, dehydration or left ventricular heart failure). This intervention could lead to earlier personalized treatment [35]. Electronic alerts for AKI can be made based on the detection of changes of a biomarker, such as serum creatinine, with the limit of basal value determinations; otherwise, eAKI can be organized on multiple markers, such as clinical, anamnestic, and biochemical markers, moreover, the implementation of a care bundle alarm can result in an improvement in outcomes [36]. The ELAIA-1 trial, which aims to ascertain the usefulness of eAKI on disease progression and mortality, is still ongoing [37].

In our study the score is calculated with an algorithm that takes into account “blocks” of urine-output/hour in a dynamic movement. The result can be summarized as *“for every six triggered alarms only one is false”*, and *“for every 12 non-triggered alarms only 2 cases are false negatives”*.

Several limitations of our study should however be mentioned.

First, this is a retrospective study based on data from hospitals in the United States.

Second, the precision of an hourly urine output report could be limited, therefore automated electronic recording appears to be required.

Third, the definition of AKI based on serum creatinine may be limited due to the nadir recognition, which in this study was the lowest recorded value during the hospital stay, and a missed AKI diagnosis could occur in this scenario. We report an incidence of AKI stage 2/3 in 3% of ICU patients, and consequently the question of selection arises. Although the incidence in the ICU varies with different definitions, based on the international FINNAKI and AKI-EPI studies (38–21), 35% to 60%, of ICU patients may be affected [38, 39]. Recently, the prospective observational study of Wiersema et al. [40], which defined AKI according to the KDIGO criteria, reported an occurrence of stage 2/3 between 3.3 and 4.5%. Uchino observed AKI in 5.7% of 29,269 critically ill patients (41). Therefore, the incidence of AKI in our population appears to be in agreement with other studies, particularly since patients undergoing hemodialysis (AKI-D) were excluded. The decision to exclude AKI-D was based on the fact that, in our opinion, the database we used did not provide sufficient information on hemodialysis.

In conclusion, urine output can correctly predict the development of oliguric Acute Kidney Injury, and the highest accuracy was obtained with a deep learning model. The characteristics of urine output in terms of dynamic flow analysis, more than a fixed volume and time, are necessary to implement the predictive score. Its applications into an e-Alert system, which is able to automatically inform the medical staff of the risk, could be useful for reducing the incidence of AKI.
